# Layer-by-Layer Insight into Electrostatic Charge Distribution of Few-Layer Graphene

**DOI:** 10.1038/srep42821

**Published:** 2017-02-21

**Authors:** Hossein Rokni, Wei Lu

**Affiliations:** 1 Department of Mechanical Engineering, University of Michigan, Ann Arbor, Michigan 48109, United States

## Abstract

In few-layer graphene (FLG) systems on a dielectric substrate such as SiO_2_, the addition of each extra layer of graphene can drastically alter their electronic and structural properties. Here, we map the charge distribution among the individual layers of finite-size FLG systems using a novel spatial discrete model that describes both electrostatic interlayer screening and fringe field effects. Our results reveal that the charge density in the region very close to the edges is screened out an order of magnitude more weakly than that across the central region of the layers. Our discrete model suggests that the interlayer charge screening length in 1–8 layer thick graphene systems depends mostly on the overall gate/molecular doping level rather than on temperature, in particular at an induced charge density >5 × 10^12^ cm^−2^, and can reliably be determined to be larger than half the interlayer spacing but shorter than the bilayer thickness. Our model can be used for designing FLG-based devices, and offers a simple rule regarding the charge distribution in FLG: approximately 70%, 20%, 6% and 3% (99% overall) of the total induced charge density reside within the four innermost layers, implying that the gate-induced electric field is not definitely felt by >4th layer.

Since its discovery in 2004, single-layer graphene (SLG) has become the most studied nanomaterial due to its exceptional mechanical^1^, electrical^2^ and optical^3^ properties. Although several physical properties are shared between SLG and few-layer graphene (FLG), increasing layer thickness can give rise to a unique range of electronic and structural properties that has not yet been sufficiently understood, in particular for FLG systems with more than 3 layers. More specifically, electrical noise, charge transport and nonlinear optical properties of FLG on substrates (usually SiO_2_/Si) exhibit strong dependence on the number of layers, gate-induced charge densities and underlying oxide substrates. It is therefore crucial in the design of FLG-based high-speed transistors^4^, terahertz plasmonics^5^, photonics and optoelectronic devices^6^ to quantitatively understand the role of the number of layers in the charge distribution and the electric field screening of the FLG/SiO_2_/Si systems and also to explore the unclear relationship between the excess gate-induced charge densities and the layer-by-layer Fermi level and charge density profiles in the FLG systems.

Owing to the importance of the subject, the question of interlayer charge screening length *λ* in the FLG systems has been addressed by several experimental methods, including angle-resolved photoemission spectroscopy (*λ* = 0.14 − 0.19 nm)^7^, nondegenerate ultrafast mid-infrared pump-probe spectroscopy (*λ* = 0.34 nm)^8^, Kelvin probe force microscopy^9^,^10^,^11^,^12^,^13^ (*λ* = 1.36 − 1.70 nm^11^, *λ* = 0.42 nm^12^ and *λ* = 2.4 nm^13^), single-gated field effect transistor (*λ* = 0.6 nm)^14^, double-gated field effect transistor (*λ* = 1.2 nm)^15^ and dark-field scattering spectroscopy (*λ* = 1.2 ± 0.2 nm)^16^. However, a relatively wide range of experimental values for *λ* (from less than a single layer to seven layers) is observed, which is not yet fully understood. Nevertheless, we believe that a part of this data scattering may be attributed to the dependence of the screening length on the device quality and experimental conditions, such as sample preparation processes, the presence of defects and impurities in graphene, the intrinsic charge density in each graphene layer and the actual doping level of the system. This diversity in the reported values of *λ* is also seen in theoretical approaches^17^,^18^,^19^,^20^. Depending on whether the inter-layer electron tunneling is taken into account or not, *λ* between 0.54 nm^17^ and 0.7 nm^18^ is obtained using a random phase approximation. Kuroda and coworkers theoretically reported that both the gate charge and temperature could highly influence *λ*, whose value may range from ~0.2 nm to 3.1 nm^19^. We will later show in this paper that the presence of the effective mass, a key missing parameter in Kuroda’s model^19^, not only leads to a much narrower range of *λ* values (=0.2 − 0.7 nm), but also rules out the possible effect of temperature on the reported values of *λ*. We also note that the *λ* value of 2.4 nm reported in ref. 13 was extracted from Kuroda’s model, while our discrete model yields a value of 0.33 nm.

Finite-size FLG flakes and graphene nanoribbons in actual devices exhibit an intriguing dependence of the electrostatic and electrical conductivity response on their geometrical parameters (e.g., lateral sizes, thicknesses, shapes and edge types)^21^,^22^. Both experimental and theoretical studies have demonstrated that a strong charge accumulation takes place at the edges of the finite-size graphene flake due to the electrostatic fringe field effects^23^,^24^,^25^,^26^,^27^,^28^,^29^,^30^,^31^. Scanning gate microscope measurements of a monolayer graphene device on a SiO_2_/Si substrate reveal significant conductance enhancement at the edge of the graphene device due to the strong charge accumulation^23^. Similar observations of inhomogeneous charge density and capacitance profiles near the edges of both suspended and hBN-supported mono/bilayer graphene devices have been reported using quantum Hall edge channels^24^,^25^. Among different theoretical models on the charge distribution of the finite-sized graphene, we particularly note a strong charge accumulation at the edges and the corners of a positively charged rectangular graphene sheet using the charge/dipole molecular dynamics model^26^,^27^ and along the edges of a graphene nanoribbon using the tight-binding model^28^.

Despite recent progress, a detailed understanding of the electrostatic charge distribution in connection with the actual electronic structure of FLG is still lacking. Here, we exploit the layered nature of FLG to develop a novel spatial discrete model that successfully accounts for both electrostatic screening and fringe field effects on the charge distribution of the finite-size FLG system. To this end, an effective bilayer model based on two tight-binding parameters is utilized to accurately describe electronic band structures and thus density of states (DOS) of one to eight Bernal-stacked graphene layers. We then explore the unclear relationship between the gate-induced charge densities and layer-by-layer Fermi level and charge density profiles in FLG systems using a global energy minimization, where its total energy is calculated based on electrostatic interaction between graphene layers and band-filling energy in each layer. Our discrete model offers a unique capability to quantify the nonlinear charge density profile, interlayer capacitance, quantum capacitance, and local surface electrostatic potential of FLG by showing a very good qualitative and quantitative agreement between the previously measured work functions in FLG and our theoretical results.

## Spatial Discrete Model

**Figure 1:**
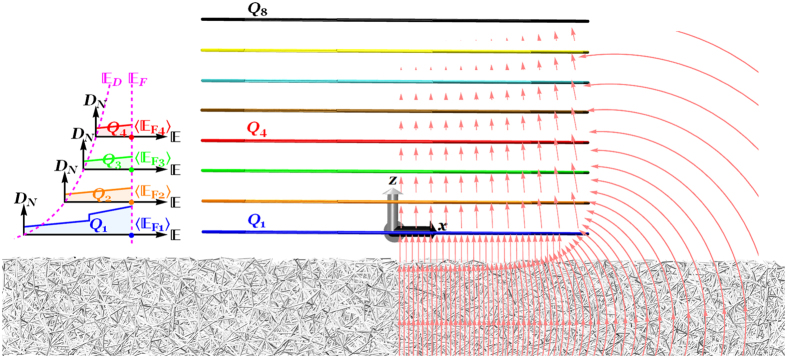
Schematic illustration of an eight-layer graphene/SiO_2_ system. The Si substrate beneath the SiO_2_ film is not shown for simplicity. The arrows correspond to the electric field lines focusing near the edges of FLG. Left inset: density of states in the four innermost graphene flakes versus the electronic band energy, where the transparent area represents the average induced charge density *Q*_*i*_ and the average value of the Fermi energy profile is denoted by 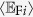.

We first examine the charge distribution of an FLG/SiO_2_/Si system containing *N* (up to 8) layers of finite-size graphene sheet with desired shapes (i.e., square, rectangle, circle or ribbon), as schematically illustrated in Fig. 1(a). Each graphene layer is labeled by an integer number starting from *i* = 1 for the layer closet to the substrate (hereafter referred to as the innermost layer) to *i* = *N* for the top layer (as the outermost layer). Applying a bias voltage *V*_0_ between the highly-doped Si substrate and *N*-layer graphene (*N*-LG) induces a total excess charge density of *Q*_0_ in *N*-LG, whose layer *i* can carry a charge density of *Q*_*i*_ such that the following constraint holds 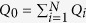.

**Figure 2:**
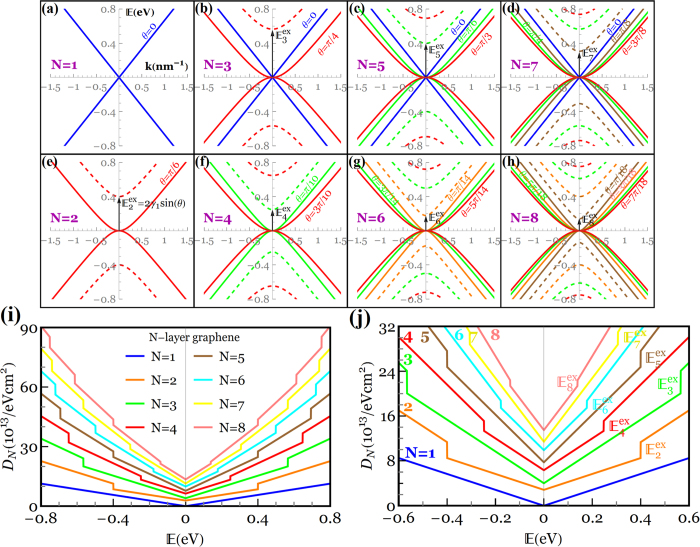
(**a**–**h**) Low‒energy band structures of Bernal‒stacked *N*‒LG near the K‒point of the Brillouin zone. There exist 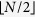 pairs of split-off hyperbolic bands, where 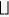 denotes the integer part of the quantity. The excitation energy from the ground state to the first excited state (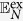) is shown with arrows. Blue lines in (**a**–**d**) correspond to the electronic dispersion of the effective monolayer graphene (*θ* = 0) which only appears in systems with an odd number of graphene layers, whereas red, green, pink and brown in (**e**–**h**) correspond to the electronic dispersion of the bilayer-like graphene (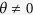). Negative and positive 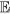 refer to the valence(hole)/conduction(electron) bands, respectively. (**i**) Density of states in *N*‒LG showing discontinuous jumps at the excited states. (**j**) Zoom-in view of discontinuous jumps at the first excited state (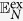).

The electronic bands of *N*‒LG can be modeled by two tight-binding parameters, namely, the nearest neighbor hopping parameter *γ*_0_ (which defines the Fermi velocity 
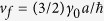
, where *a* = 0.142 *nm* is the C-C bond length) and the nearest neighbor interlayer coupling constant *γ*_1_. We take *γ*_0_ = 3.14 *eV* and *γ*_1_ = 0.4 *eV* as typical values of bulk graphite. The energy dispersion in Bernal-stacked *N*‒LG, obtained from 2D cuts in the electronic dispersion of graphite, perpendicular to the graphene planes at specific values of *θ* = *jπ*/2(*N* + 1), can be given by 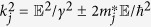, where *γ* = *v*_*f*_*ħ (ħ* being the reduced Planck constant), 
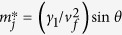
 is the effective mass, *j* (=1, 3, 5, …, *N* − 1 for even layers and 0, 2, 4, …, *N* − 1 for odd layers) is the index of the energy band with kinetic energy 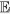. Figure 2(a) through (h) illustrate low‒energy band structures of *N*‒LG (near the K‒point of the Brillouin zone) up to *N* = 8. It is seen that monolayer graphene (Fig. 2(a)) exhibits a well-known linear dispersion which results in massless excitations, whereas bilayer graphene (Fig. 2(e)) displays a set of four hyperbolic bands (with no Dirac electrons) touching at the so-called Dirac point. Though the band structure of trilayer graphene (Fig. 2(b)) comprises one pair of linear (monolayer-like) bands and two pairs of hyperbolic (bilayer‒like) bands, tetralayer graphene (Fig. 2(f)) interestingly shows only four pairs of hyperbolic (bilayer‒like) bands. In general, based on the tight-binding model described above, both monolayer‒ and bilayer‒like bands are present in odd multilayers (*N* ≥ 3), whereas the band structure of even multilayers only consists of the bilayer‒like bands. Figure 2(a–h) confirm that *N*‒LG should be considered a single 2D system (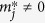), rather than a composite system consisting of *N* parallel single layers of graphene with the linear energy dispersion (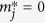), as experimentally confirmed by micro magneto-Raman scattering spectroscopy in 1- to 5-LG systems^32^. We will address at the end of the paper the influence of the effective mass on the charge distributions of the *N*‒LG system through comparison of our results with those obtained by a massless linear energy dispersion model.

The density of states (DOS) in *N*-LG is obtained from the summation of the DOS for each energy band with double spin and double valley degeneracies


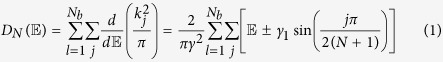


where *N*_*b*_ (=*N*/2 and (*N* + 1)/2 for even and odd multilayers, respectively) is the number of bands in 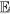 and *j* = 2*l* − 1 and 2(*l* − 1) for even and odd multilayers, respectively. A systematic evolution of 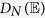 as a function of the layer number in Fig. 2(i) reveals finite discontinuities at the split-off (excitation) energies 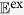 (=2*γ*_1_ sin *θ*) which are produced by the band extrema at the K-point, followed by a linear increase with kinetic energy 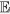. Of particular importance for the electronic structures of *N*-LG at low energies is the excitation energy from the ground state (Dirac point) to the first excited state (denoted by 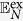), as explicitly shown in Fig. 2(j).

We next determine the charge distribution profile in a finite-size *N*-LG stack with a circular shape of radius *R*, based on the method of images, followed by solving the Love equation (Section S1.1 of Supplemental Material (SM)). The charge density profile in the circular layer *i* can then be expressed by


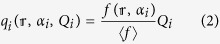


where


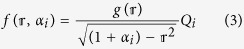


is the charge distribution profile, normalized to its average value 〈*f*〉 for generality purposes; the index notation *i* varies from 1 to *N*; 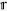 (=*r*/*R*) is a dimensionless parameter; *r* denotes the radial coordinate of atom and 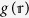 is a polynomial function of 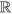 which only depends on the ratio of the graphene size to the dielectric thickness (Fig. S1 of SM). A new parameter *α*_*i*_ (>0) is introduced by Eq. (3) in order to determine the amount of charge density at the edge of the layer *i* (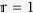). Although the focus of the present work is on graphene flakes with a circular shape, we note that the charge distribution of circular graphene flakes and graphene nanoribbons is of a similar form as given by Eq. (3) and, therefore, does not qualitatively and pretty much quantitatively alter the main results of this paper (Section S1.2 of SM). We also refer the interested reader to Section S1.3 of SM for the corresponding charge distribution profile of rectangular/square graphene flakes.

As we already discussed, in practice, the charge distribution in electrostatically doped graphene devices is inhomogeneous, yielding a non-uniform Fermi level profile. For instance, scanning gate microscope measurements of a monolayer graphene device on a SiO_2_/Si substrate reveal a strong shift of the local Dirac point from the Fermi level at the graphene edge due to the contribution of both localized edge states (i.e., zigzag or armchair) and accumulated charge along the edge^23^. The Fermi energy profile 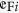 across the layer *i* can be expressed in terms of the constant Fermi energy 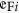 as follows (Section S2 of SM)


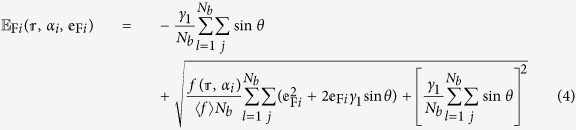


Then, the average charge density of each layer can be expressed by


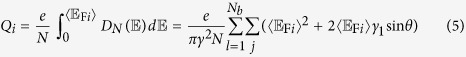


where 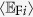 is the average value of 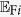 in terms of 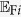 and *α*_*i*_. The average charge density *Q*_*i*_ can be obtained by minimizing the total energy of the system with respect to 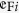 and *α*_*i*_ as the variational parameters under the constraint that 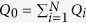. In the *N*-LG/SiO_2_/Si system, the total energy can be split as, *U*_*t*_ = *U*_*r*_ + *U*_*e*_ + *U*_*b*_, where the terms correspond to energy stored in SiO_2_ as the dielectric medium (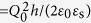 where *h* and *ε*_s_ are the SiO_2_ thickness and the dielectric constant, respectively, and *ε*_0_ is permittivity of the vacuum), electrostatic energy between the graphene layers and the band-filling energy in each layer, respectively. Charge distribution in the *N*-LG system can be explained as a result of the competition between *U*_*e*_ that tends to hold the charge in the layers as close to the Si substrate as possible, and *U*_*b*_ that tends to spread the charge throughout the *N*-LG system. Assuming that the electronic band structures remain unchanged under an external electric field, *U*_*e*_ and *U*_*b*_ at zero temperature can be given, respectively, by


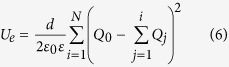


and


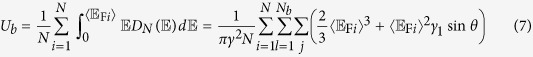


where *d* is the interlayer distance and *ε* is the dielectric constant in *N*-LG. For our numerical calculations, we use the value *ε* = 1, which describes the *N*-LG system in vacuum. One may find the equivalent bias voltage applied between the Si substrate and *N*-LG by taking the derivative of the total energy with respect to the total induced charge density (i.e., *V*_0_ = *dU*_*t*_/*dQ*_0_) and local surface electrostatic potential of each layer can be obtained by *V*_*i*_ = *dU*_*e*_/*dQ*_*i*_.

## Results and Discussion

### Comparison Studies

**Figure 3:**
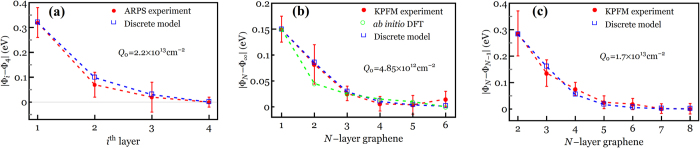
(**a**) Work functions across a 4-LG system which are given relative to that of the outermost layer 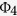 as the zero-reference level for *Q*_0_ = 2.2 × 10^13^ cm^−2 ^7; (**b**) work functions in the 1-6-LG systems relative to that of bulk graphite 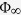 for *Q*_0_ = 4.85 × 10^12^ cm^−2 ^11; and (**c**) difference between the work function of the uppermost layer in the *N*-LG system and that in the (*N*-1)-LG system for *N* = 1 to 8 when *Q*_0_ = 1.7 × 10^13^ cm^−2 ^12.

In order to verify the accuracy of the results presented in this paper, we first compare our local work functions (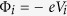) with those measured by angle-resolved photoemission spectroscopy (ARPS)^7^ and Kelvin probe force microscopy (KPFM)^11^,^12^. We note that since the accurate work function of the tip under the ambient conditions and also the accurate value of the dielectric constant for the *N*-LG/SiO_2_ interface are unknown, the difference of the work function is used to achieve more accurate comparison purposes. We begin by comparing 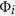 in a 4-LG system with that measured by ARPS^7^, as shown in Fig. 3(a). The results are given relative to the work function of the outermost layer 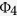 as the zero-reference level and *Q*_0_ is set to be 2.2 × 10^13^ cm^−2^. It is evident from Fig. 3(a) that a very good agreement exists between the proposed discrete model and those measured by Ohta *et al*.^7^. Another comparison study is conducted in Fig. 3(b) between the present discrete model and KPFM results of Ziegler *et al*.^11^, who measured 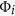 in the 1-6-LG systems relative to that of bulk graphite 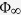. Figure 3(b) clearly demonstrates that the measured work functions are generally in much better agreement with our results than those obtained by *ab initio* DFT calculations^11^ when assuming a total induced charge density of 4.85 × 10^12^ cm^−2^. We further perform a similar comparison in Fig. 3(c) between the present work functions at the uppermost layer of *N*-LG (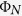) relative to those of (*N* − 1)-LG (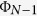) with KPFM results measured for *N*-LG with layer number ranging from 1 to 8^12^. It is indicated that the present work functions closely match with the experimental observations for *Q*_0_ = 1.7 × 10^13^ cm^−2^.

**Figure 4:**
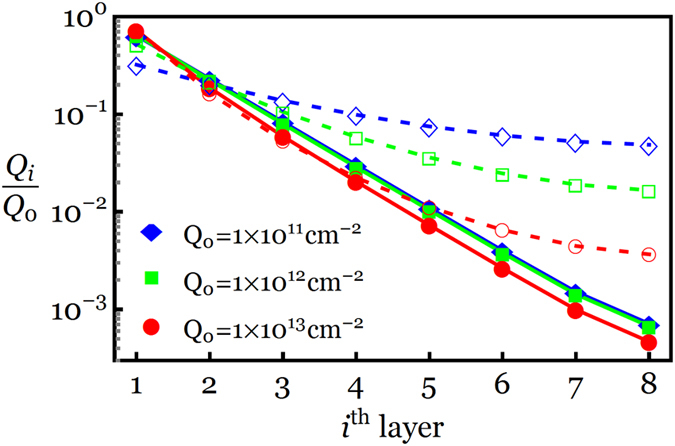
Normalized charge distribution profiles of an 8-LG system for three different values of *Q*_0_. Dashed curves with open symbols represent the results obtained by the linear energy dispersion (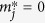), whereas solid curves with filled symbols denote the results obtained by the actual energy dispersion of an 8-LG system (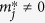).

Further comparison study is performed in Fig. 4 to investigate the influence of the effective mass 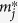 on the charge distribution of an 8-LG system. It is seen from Fig. 4 that the model based on the monolayer-like band structure fails to accurately predict the charge distribution of the 8-LG system, in particular at the smaller induced charge densities. This figure also shows a significant deviation in the charge densities of layers *i* > 5 for *Q*_0_ = 10^13^ cm^−2^.

Also, our energy evaluations of *N*-LG systems under a given *Q*_0_ for three possible charge distribution scenarios- (a) optimum distribution given in Eq. (3) non-uniform distribution with the charge singularity at the very edge (i.e., *α*_*i*_ = 0), and (c) fully uniform distribution (i.e., *q*_*i*_ = *Q*_*i*_)- reveal that the minimum energy is only achieved by the present optimum charge distribution model, further indicating its merit in predicting the charge distribution of other families of atomically thin layered materials.

### Layer‒by‒Layer Charge Density Profiles in 5‒LG System

**Figure 5:**
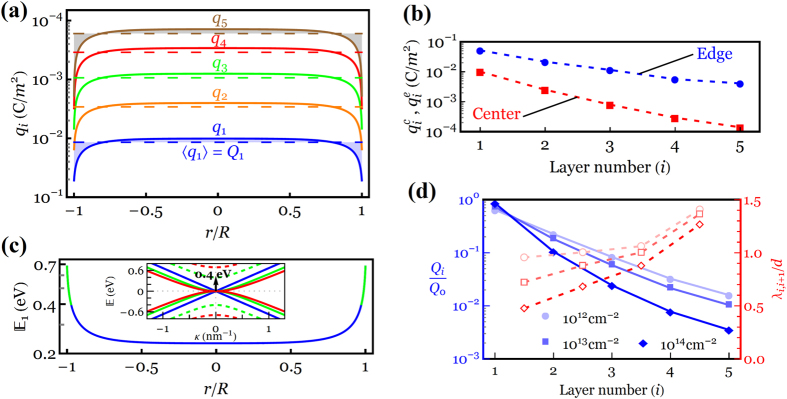
(**a**) Charge density profiles of a 5-LG system for *Q*_0_ = 10^13^ cm^−2^, where each dashed line represents the average charge density 〈*q*_*i*_〉 = *Q*_*i*_ in the layer *i*. (**b**) Charge density at the edge 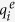 and the center 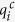 of the layer *i*. (**c**) Fermi level profile of the innermost layer. Inset: low-energy band structure of 5-LG system. Solid green curve in the Fermi level profile and dashed green curve in the band structure represent the first (0.4 eV) excitation energy. (**d**) Blue curves: normalized average charge profiles across the layers of a 5-LG system for different gate charge densities of 10^12^ (circles), 10^13^ (rectangles) and 10^14^ cm^−2^ (diamonds). Red curves: corresponding changes in the local charge screening *λ*_*i*,*i*+1_.

We now explore the unclear relationship between the total induced charge densities and the layer-by-layer charge density and Fermi level profiles. To this end, we begin by illustrating the charge density profiles of the 5-LG system when *Q*_0_ = 10^13^ cm^−2^, as shown in Fig. 5(a) (see Fig. S2(a) in the SM for the corresponding Fermi level profiles). Consistent with the experiments of Ohta *et al*.^7^ and Wang *et al*.^12^, the charge density is drastically reduced as one move away from the innermost toward the outermost layer. However, the charge density in the region very close to the edges is screened out an order of magnitude more weakly than that across the central region of the layer, as shown in Fig. 5(b), which can be explained by the presence of the strong fringe field along the edges, as schematically shown in Fig. 1. Our results in Fig. 5(a) also suggest that the innermost layer plays the most important role in the electrostatic charge distribution of the *N*-LG systems by hosting ~70% of the gate charge density *Q*_0_. Hence, it is worth looking into its Fermi level profile more in detail, as illustrated in Fig. 5(c). By following the evolution of the Fermi level along the innermost layer, it is observed that a strong charge accumulation and thus sufficiently large shift in the Fermi energy at the edge can give rise to a jump in the electronic band structures of 5-LG toward the first excited state, 0.4 eV (as shown in green solid curve in Fig. 5(c) and in green dashed curve in the inset, which shows the energy band structure of the 5-LG system). However, our Fermi level analyses in the innermost layer of 6-and 8-LG systems exhibit few jumps in the Fermi level of the regions both close to and away from the edges when *Q*_0_ = 10^13^ cm^−2^ (see Fig. S2(b) of SM for detailed discussions). This can be attributed to the fact that the lowest energy of the first excitation band decreases for the *N*-LG system with a larger number of graphene layers, as shown in Fig. 5(b).

To quantitatively elucidate the correlation between the magnitude of the gate charge density *Q*_0_ and the average charge distribution *Q*_*i*_ through the 5-LG thickness, Fig. 5(d) shows *Q*_*i*_/*Q*_0_ ratio as a function of the layer positions for three different values of *Q*_0_ (=10^12^, 10^13^ and 10^14^ cm^−2^). It is seen that a larger value of *Q*_0_ leads to a stronger charge screening normal to the layers, however, this effect diminishes when *Q*_0_ < 10^12^ cm^−2^. This figure also demonstrates that almost 90% of the excess charge density resides in the first two layers, implying that the interlayer screening length can reliably be determined to be less than ~0.7 nm. Having *Q*_*i*_ data for each layer enables us to calculate the “local” (interlayer) charge screening *λ*_*i*,*i*+1_ as 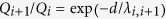 based on Thomas-Fermi charge screening theory (see Section S4 of SM for the calculation of the interlayer screening). It is deduced from Fig. 2(d) that the charge screening length between the first and second layers *λ*_1,2_ may reduce from ~1*d* at *Q*_0_ = 10^12^ cm^−2^ to ~0.5*d* at *Q*_0_ = 10^14^ cm^−2^, while a smaller variation in *λ*_*i*,*i*+1_ is observed for the layers farther from the substrate due to the reduction in their DOS at the Fermi level.

### Layer‒Dependent Charge Screening in *N*‒LG Systems

**Figure 6:**
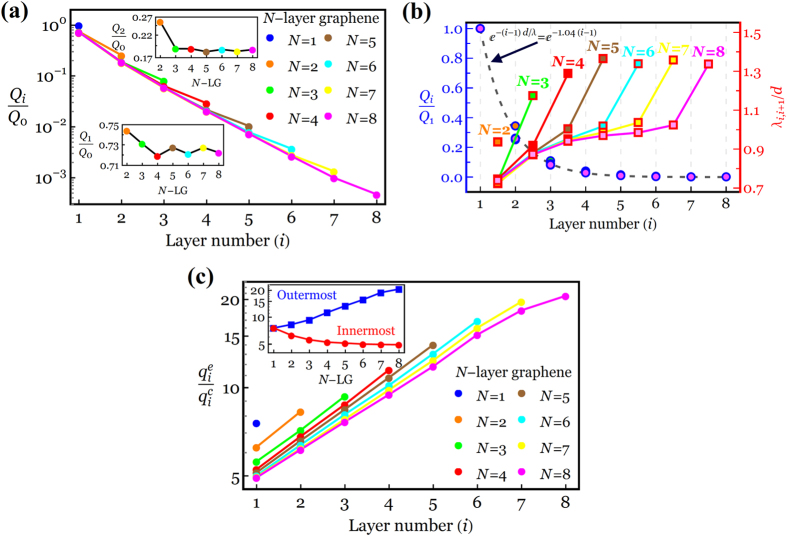
(**a**) Normalized average charge distribution profiles across the layers of 1-8-LG systems for *Q*_0_ = 10^13^ cm^−2^. Insets: Normalized charge density of the first (lower inset) and second (upper inset) layer in 2-8-LG. (**b**) Circles with blue borders: global charge screening length in 1-8-LG systems for *Q*_0_ = 10^13^ cm^−2^. A decay length (*d*/*λ*) of 1.04 is found by fitting the data with a function *e*^−(*i*−1)*d*/λ^, indicated by a dashed curve. Rectangles with red borders: local charge screening length in 1-8-LG systems for *Q*_0_ = 10^13^ cm^−2^. (**c**) Edge-to-center charge density ratio as a function of the layer position in 1-8-LG systems when *Q*_0_ = 10^13^ cm^−2^. Inset: Edge-to-center charge density ratio for the innermost (red circles) and outermost (blue squares) layers of 1-8-LG systems.

We now turn to a discussion of the layer-dependent charge distribution/charge screening in 1-8-LG systems for a given gate-induced charge density of 10^13^ cm^−2^. Figure 6(a) presents a plot of *Q*_*i*_/*Q*_0_ *versus* the layer positions in 1-8-LG systems, indicating that approximately 70%, 20%, 6% and 3% (99% overall) of *Q*_0_ sit in layers *i* = 1 to 4, respectively, and thus the gate-induced electric field is not definitely felt by *i* > 4 layers. Interestingly, we observed that the charge density of the layers located in the same position in *N*-LG systems decreases in a sawtooth-like fashion, as shown in the insets of Fig. 6(a) for the normalized charge density of the innermost *Q*_1_/*Q*_0_ and second innermost *Q*_2_/*Q*_0_ layers. This saw-tooth pattern which is associated with the presence of the linear energy dispersion in *N*-LG with odd layer number has been experimentally confirmed through the measurement of the electric double-layer capacitance between an ionic liquid and 1-6-LG^33^. The results in Fig. 6(a) provide an important piece of information about the charge screening effect of the innermost layer on different layers of 2-8-LG. Hence, we first define a “global” (effective) charge screening *λ* as *Q*_*i*_/*Q*_1_ = exp[−*d*(*i* − 1)/*λ*]. This new definition of the “global” charge screening length allows us to explore how the innermost layer impacts the surface potential drop across the FLG thickness and also provides a single value of the screening length to predict the charge distribution of all layers relative to that of the innermost layer. Keeping both global and local screening definitions in mind, we observe from Fig. 6(b) that our global charge screening can be well fitted by the simple exponential decay function (in particular for *Q*_0_ ≤ 10^13^ cm^−2^, see Fig. S3 of SM) when 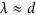. Figure 6(b) also illustrates the local charge screening between the adjacent layers of 1-8-LG, showing a much lower variation in *λ*_*i*,*i*+1_ of the middle layers with an average value of ~*d*, consistent with the global charge screening length. It is also observed from Fig. 6(b) that *λ*_*i*,*i*+1_/*d* of the innermost and outermost interlayers becomes layer-independent for *N* ≥ 3 and *N* ≥ 4, respectively.

We next address the problem of the charge accumulation along the graphene edge, focusing first on very limited publications that have *quantitatively* studied the charge density at the edge of graphene thus far. From prior experimental work, a nearly three-fold increase in capacitance and thus the charge density near the edge of a suspended bilayer flake (0.4 *μ*m wide and 2.6 *μ*m long) was observed using quantum Hall edge channels^24^. From theoretical points of view, the charge/dipole molecular dynamics model predicts a seven-fold (fifteen-fold) enhancement of the charge density at the edge (corner) over that at the center of a charged 8.5 nm × 4.8 nm rectangular graphene sheet^26^ and a similar eight-fold enhancement of the charge density in a 20-nm-wide graphene nanoribbon^27^. This model also suggests that the charge enhancement is more significant in multi-layered graphene in such a way that the charge density at the edge relative to that at the center can vary from 9 in the inner layer to >14 in the outer layer of a 4-LG nanoribbon system^27^. Also, using the tight-binding Hartree model, the charge density along the edge of a 20-nm-wide graphene nanoribbon enhances up to five times over that at the center^28^.

Having this quantitative description of the charge accumulation at the graphene edge in mind, we present in Fig. 6(c) the charge density at the edge relative to that at the center, 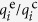, as a function of the layer position in the 1-8-LG systems for *Q*_0_ = 10^13^ cm^−2^. As is evident from the figure, our discrete model predicts the edge-to-center charge density ratio for monolayer graphene to be ~7.5 which is consistent with the theoretical results^26^,^27^,^28^. Surprisingly, the addition of each extra layer reduces the charge accumulation at the edge of the innermost layer from 7.5 in 1-LG down to ~5 in 8-LG, whereas an inverse trend is observed for the charge accumulation at the edge of the outermost layer, whose value varies from 7.5 in 1-LG up to ~20 in 8-LG, as shown in the inset of Fig. 6(c). While the latter can be attributed to the presence of highly weak charge screening at the edge due to the strong fringe field effect, as already shown in Fig. 5(b), the former may be accounted for by a combined effect of strong repulsive forces at the edge and the overall charge reduction in the innermost layer. It is worth pointing out that such reduction of the charge accumulation at the edge is observed in all other layers having the same position in the *N*-LG systems (for instance, see the second innermost layer in 2-8-LG) and the edge-to-center charge density ratio eventually converges to a constant value, showing nearly layer-independent behavior for *N* ≥ 6.

### Temperature‒Dependent Charge Screening Model

**Figure 7:**
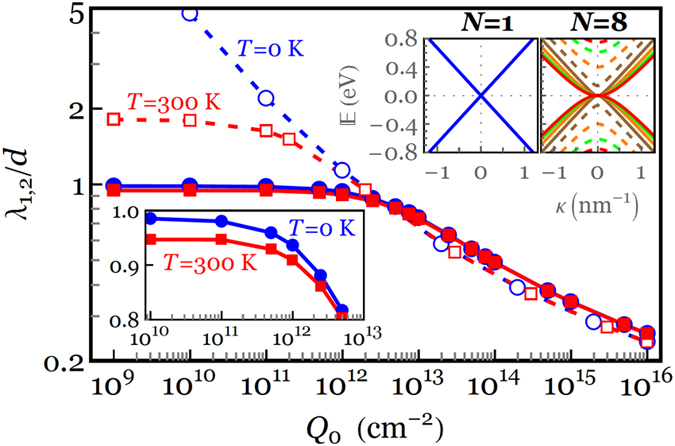
Local screening length between the first and second layers of an 8-LG system as a function of *Q*_0_. Dashed curves with open circles (squares) represent the results obtained by the linear energy dispersion model (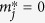) at *T* = 0 K (*T* = 300 K), whereas solid curves with filled circles (squares) denote the results obtained by the actual energy dispersion of the 8-LG system (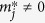) at *T* = 0 K (*T* = 300 K).

While the present study has focused on the charge distribution of *N*-LG at absolute zero temperature, we note that a variation in temperature from zero to room temperature has no appreciable effect on the charge screening length, more specifically at the higher gate electric field. Following a temperature-dependent model of the charge distribution detailed in Section S5 of SM, the local charge screening between the first and second layers of an 8-LG system is plotted in Fig. 7 as a function of *Q*_0_ at *T* = 0 and 300 K. For comparison purposes, the results of Kuroda *et al*.^19^ based on the linear energy dispersion are reproduced by setting 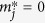, as indicated by dashed curves with open symbols in Fig. 7. It is evident from Fig. 7 that the interlayer charge screening is insensitive to the temperature variation when *Q*_0_ ≥ 5 × 10^12^ cm^−2^ and only a slight change in *λ*_1,2_ is observed at smaller gate charge densities (see lower inset) and ultimately saturates to 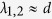. Consistent with our temperature-independent charge screening length, Yang and Liu reported using the first-principles calculations that the interlayer screening, static perpendicular dielectric function and density of states of bi- and tri-layer graphene slightly changes as temperature increases from 0 K to 300 K to 600 K^34^. It is also observed from Fig. 7 that the linear dispersion model fails to predict the interlayer charge screening between the two innermost layers for 
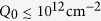
 such that *λ*_1,2_ goes to infinity (i.e. 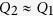) at *T* = 0 as *Q*_0_ → 0. Interestingly, a layer-by-layer inspection of the charge density in a similar 8-LG system for different values of *Q*_0_ reveals that the linear dispersion model not only yields inconsistent charge density profiles in almost all layers for 
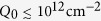
 but also shows a significant deviation in the charge densities of outer layers for *Q*_0_ > 10^12^ cm^−2^, as shown earlier in Fig. 4. This deviation from our model can be understood in terms of the effective mass in *N*-LG with *N* ≥ 2: an essential ingredient that is not captured in Kuroda’s model where an *N*-LG system is considered as *N* parallel single layers with a massless linear energy dispersion (upper inset for *N* = 1), rather than a single 2D system with the actual energy dispersion (upper inset for *N* = 8).

## Conclusions

We developed a novel spatial discrete model to unravel the relationship between the macroscopic induced charge density and microscopic (layer-by-layer) charge distribution in finite-size FLG through considering the effects of both electrostatic interlayer screening and fringe field. We showed that adding each extra layer reduces the charge accumulation at the edge relative to that at the center of the innermost layer up to 20% (from ~7.5 in 1-LG down to ~5 in 8-LG). Our model offers a simple rule of thumb regarding the charge distribution in FLG: approximately 70%, 20%, 6% and 3% (99% overall) of the total induced charge density reside within the four innermost layers (layers *i* = 1 to 4, respectively), implying that the gate-induced electric field is not definitely felt by layers *i* > 4. We finally found that a variation in temperature from zero to 300 K has no appreciable effect on the interlayer charge screening when the gate charge density is larger than ~5 × 10^12^ cm^−2^. Although our study is concerned with FLG systems, the generality of our spatial discrete model suggests that the charge density profile, interlayer screening, quantum capacitance, and local surface potential of other atomically thin layered materials (ATLMs), such as semiconducting transition metal dichalcogenides (e.g., MoS_2_, WSe_2_ and WS_2_) and heterostructures (e.g., graphene/MoS_2_ and MoS_2_/WSe_2_), can be characterized by feeding relevant electronic band structures of ATLMs into our model. In addition, the effect of structural defects (e.g., vacancies, adatoms, dislocations and grain boundaries) and stacking faults on the charge distribution of defective FLG systems can be studied by modifying DOS of pristine FLG.

## Additional Information

**How to cite this article:** Rokni, H. and Lu, W. Layer-by-Layer Insight into Electrostatic Charge Distribution of Few-Layer Graphene. *Sci. Rep.* **7**, 42821; doi: 10.1038/srep42821 (2017).

**Publisher's note:** Springer Nature remains neutral with regard to jurisdictional claims in published maps and institutional affiliations.

## Supplementary Material

Supplemental Material
