# Supplementary material for: Layer-by-Layer Insight into Electrostatic Charge Distribution of Few-Layer Graphene
Source: Sci Rep. 2017 Feb 21;7:42821. doi: 10.1038/srep42821 (PMC5318858; doi:10.1038/srep42821)
Supplement: Supplemental Material [file srep42821-s1.doc]

# Supplemental Material for “Layer-by-Layer Insight into Electrostatic Charge Distribution in Few-Layer Graphene”

**Hossein Rokni and Wei Lu^*^**

Department of Mechanical Engineering, University of Michigan, Ann Arbor, Michigan 48109, United States

^*^ Corresponding author: [weilu@umich.edu](mailto:weilu@umich.edu)

Table of Contents

[S1. Electrostatic Fringe Field Effects in Graphene Flake 2](#__RefHeading___Toc466112350)

[*S1.1. Circular Graphene Flake* 2](#__RefHeading___Toc466112351)

[*S1.2. Graphene Nanoribbon* 3](#__RefHeading___Toc466112352)

[*S1.3. Rectangular/Square Graphene Flakes* 4](#__RefHeading___Toc466112353)

[S2. Non-Uniform Fermi Energy Profile 4](#__RefHeading___Toc466112354)

[S3. Fermi Level Profiles in *N*-Layer Graphene 5](#__RefHeading___Toc466112355)

[S4. Local and Global Interlayer Charge Screening 5](#__RefHeading___Toc466112356)

[S5. Temperature-Dependent Discrete Model 6](#__RefHeading___Toc466112357)

[References 7](#__RefHeading___Toc466112359)

## S1. Electrostatic Fringe Field Effects in Graphene Flake

## *S1.1. Circular Graphene Flake*

Both experimental and theoretical studies have demonstrated that a strong charge accumulation takes place at the edges of the finite-size graphene flake due to the electrostatic fringe field effects [1-6]. Scanning gate microscope measurements of a monolayer graphene device on a SiO_2_/Si substrate demonstrate that the charge accumulation at the edge of the graphene devices is significant, in particular in narrow devices such as graphene nanoribbons [5,6]. In addition, the charge distribution in a positively charged graphene sheet was studied using a charge/dipole molecular dynamics model and a strong charge accumulation was observed along the edges and at the corners of the rectangular graphene sheet [3,4].

In order to figure out how the induced charge is distributed within the graphene sheet under an external electric field, we consider a circular graphene sheet of radius
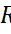
 placed at a distance of
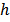
 above SiO_2_ film and at a potential
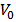
 relative to the Si substrate. The corresponding induced charge density
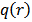
 can be calculated using the method of images. The charge density depends on the radial coordinate across the sheet, which varies in the range
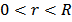
. Using the method of images, this problem is equivalent to two parallel circular sheets of radius
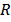
 vertically separated by a distance
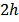
 and placed at potentials
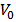
 (upper sheet) and
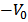
 (lower sheet), such that the Si substrate plane (
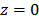
) is at zero potential. The center of the upper sheet is taken to be at
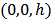
 and the center of the lower sheet at
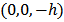
. Following the analytical solution developed by Felderhof [7], the electrostatic potential in the upper sheet can then be expressed by

| 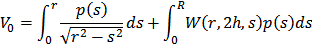 | (S1) |
| --- | --- |

where

| 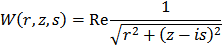 | (S2) |
| --- | --- |

satisfies Laplace’s equation (
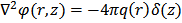
) everywhere, except on a disk of radius
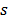
 centered at the origin in the
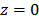
 plane, and the weight function
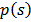
 satisfies the Love equation

| 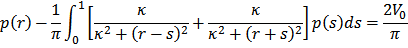 | (S3) |
| --- | --- |

where
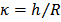
. We solved this integral equation numerically in MATHEMATICA using iteration with a suitable initial function. In order to evaluate the convergence of our numerical solution, we compared the “scaled” capacitance (multiplied by the factor
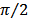
) of the sheet given by

| 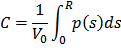 | (S4) |
| --- | --- |

with that of Cooke [8]. We obtained the scaled capacitance of 1.8208 (For
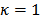
) and 9.2328 (for
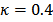
), which are in excellent agreement with those of Cooke [8] who reported the values of 1.8208 and 9.2330, respectively.

Finally, the induced charge density and the weight function are related by

| 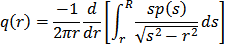 | (S5) |
| --- | --- |

In **Fig. S1**, we show the normalized induced charge density profiles (from which the Fermi energy profiles shown in **Fig. S2** are extracted) for different values of
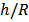
.

| 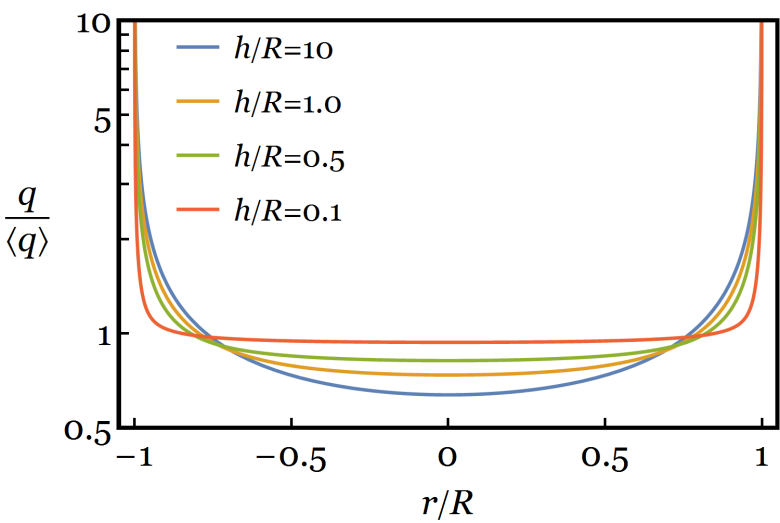  **SiO_2_**  **Si**  **2*R***  ***h***  ***V=V_0_***  ***V*=0** |
| --- |
| **Figure S1.** Induced charge density profile of the sheet for different thickness-to-radius ratios. |

In this paper, for more practical applications, we set
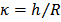
 to be 0.1 and thus the corresponding charge density profile can be given by

| 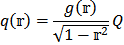 | (S6) |
| --- | --- |

where
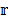
 (
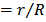
) is a dimensionless parameter,
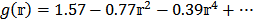
, and
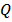
 is the total charge density.

## *S1.2. Graphene Nanoribbon*

Similar to the charge distribution profile in the circular FLG, the charge distribution in a graphene nanoribbon of width
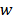
 can be given by [1]

| 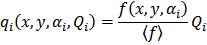 | (S7) |
| --- | --- |
| where |  |
| 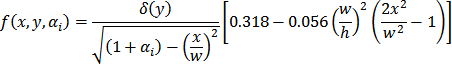 | (S8) |

where
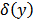
 is the delta function and
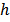
 is the dielectric thickness. The terms in the brackets are valid when the ribbon width is much smaller than the dielectric thickness (
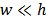
). We refer the interested reader to the supplementary material of Ref. [9] for the charge distribution of the graphene nanoribbon with different ribbon width-dielectric thickness ratios
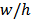
.

## *S1.3. Rectangular/Square Graphene Flakes*

Similar to the charge distribution profile in the circular FLG, the charge distribution in the rectangular graphene flakes with length
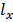
 and width
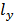
 can be given by

| 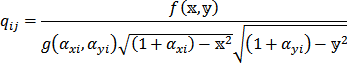 | (S9) |
| --- | --- |

where the charge distribution profile is normalized by

| 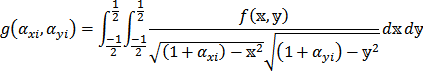 | (S10) |
| --- | --- |

where
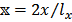
 and
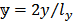
 are dimensionless parameters;
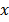
 and
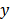
 denotes, respectively, the *x* and *y* coordinates of atom *j* in the *i*th layer which carries the corresponding charge of
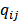
; and
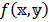
 is a polynomial function of
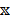
 and
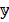
 which only depends on the ratio of the graphene size to the dielectric thickness [9]. Also,
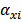
 and
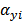
 denote the amount of charge accumulation at the middle of the *x* and *y* edges relative to that at the center of the graphene flake, respectively. From **Eq. (S9)**, the amount of charge accumulation at the corner relative to that at the center of the rectangular graphene flake is obtained to be ~
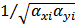
.

## S2. Non-Uniform Fermi Energy Profile

The uniform charge density of the
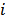
th layer is related to its corresponding constant Fermi energy
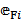
 by

| 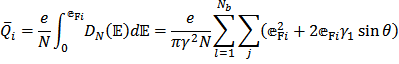 | (S11) |
| --- | --- |

Solving Eq. (S11) for
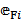
 yields

| 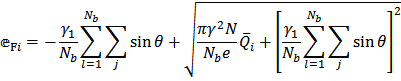 | (S12) |
| --- | --- |

Similarly, the charge density profile can be related to the fermi energy profile as follows

| 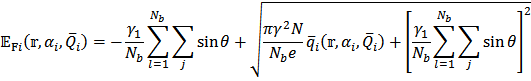 | (S13) |
| --- | --- |

defining the charge density profile by

| 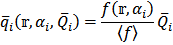 | (S14) |
| --- | --- |

and substituting **Eq. (S11)** into **Eq. (S14)** yield

| 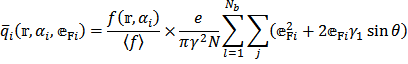 | (S15) |
| --- | --- |

Finally, substituting **Eq. (S15)** into **Eq. (S13)** leads to

| 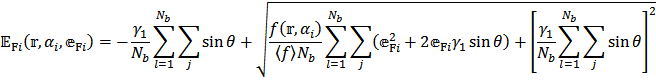 | (S16) |
| --- | --- |

## S3. Fermi Level Profiles in *N*-Layer Graphene

**Fig. S2(a)** demonstrates the Fermi level profiles of a 5-LG system, while Fermi level profile of the innermost layer in an 8-LG system is shown in **Fig. S2(b)** for
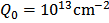
. Far from the edge, we observe from **Fig. S2(b)** that sitting ~70% of the total induced charge in the innermost layer would cause a shift in the Fermi level from the ground state to the first excited state (as shown in brown solid curve in **Fig. S2(b)** and in brown dashed curve in the inset, which shows the energy band structure of the 8-LG system). However, our Fermi level analyses in the innermost layer of the bi-, tri-, tetra- and penta-LG systems do not exhibit any jump in the Fermi level of the region away from the edge when
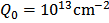
. This can be attributed to the fact that the lowest energy of the first excitation band decreases for the *N*-LG system with a larger number of graphene layers. Following the evolution of Fermi level along the innermost layer in **Fig. S2(b)**, it is observed that a strong charge accumulation and thus sufficiently large shift in Fermi energy at the edge can give rise to a jump in the electronic band structures of FLG toward the second(0.4eV) and third(0.61eV) excitation energies, as shown in orange and green curves, respectively.

| 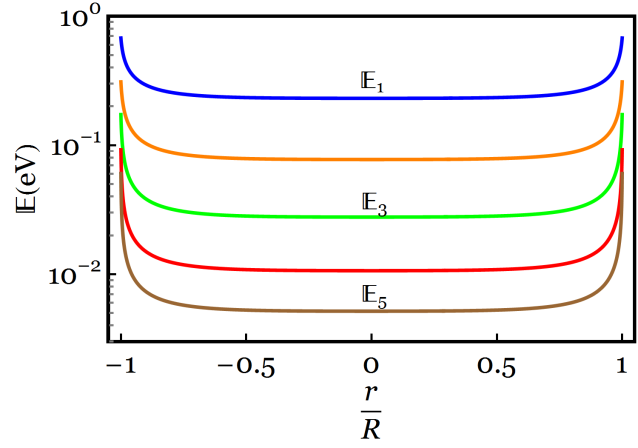  **(b)**  **(a)** | 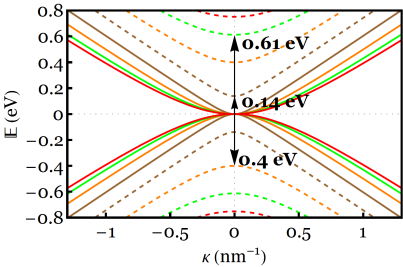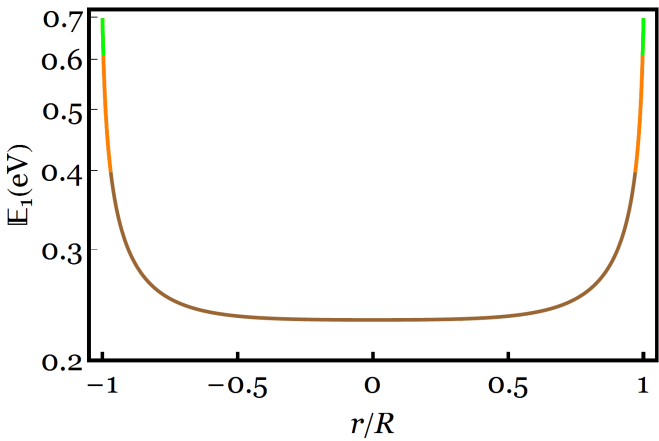 |
| --- | --- |
| **Figure S2. (a)** Fermi level profiles of a 5-LG system for 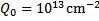. **(b)** Fermi level profile of the innermost layer in an 8-LG system for 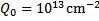. Inset: low- and high-energy band structure of the 8-LG system. Brown, orange and green solid curves in the Fermi level profile and brown, orange and green dashed curves in the band structure represent the first (0.14 eV), second (0.40 eV) and third (0.61 eV) excitation energies, respectively. | |

##

## S4. Local and Global Interlayer Charge Screening

Sui and Appenzeller [14] presented a systematic experimental study on charge and current distribution in FLG field-effect transistors and then proposed the following charge distribution in the FLG systems based on the Thomas-Fermi (TF) charge screening theory

| 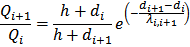 | (S17) |
| --- | --- |
| 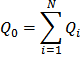 | (S18) |

where the index *i* takes on values from 1 to *N* (*N* being the total number of graphene layers); *Q*_1_ is the total induced charge in the closest layer to the SiO_2_ substrate;
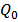
 is the total induced charge in the graphene system;
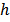
 (typically in the range of 50-300 nm) is the SiO_2_ thickness;
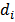
 is the distance from the bottom of the FLG system to the layer
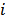
 (hence,
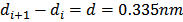
 is the distance between adjacent graphene layers); and
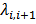
 is the local screening length between two consecutive layers. Considering that in many graphene-based electronic devices, the SiO_2_ film is much thicker than the FLG, Eq. (S17) reduces to:

| 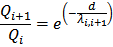 | (S19) |
| --- | --- |

We also define the global screening length
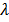
 between the innermost layer and the other layers by

| 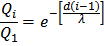 | (S20) |
| --- | --- |

**Fig. S3(a)** shows
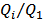
 ratio as a function of the layer positions for a 5-LG system under three different gate densities of
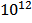
,
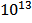
 and
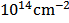
 and a similar plot for an 8-LG system for
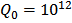
,
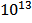
 and
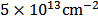
 is presented in **Fig. S3(b)**. We observe from **Fig. S3** that our data can be well fitted by an exponential decay function when
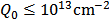
.

**(a)**

**(b)**

| 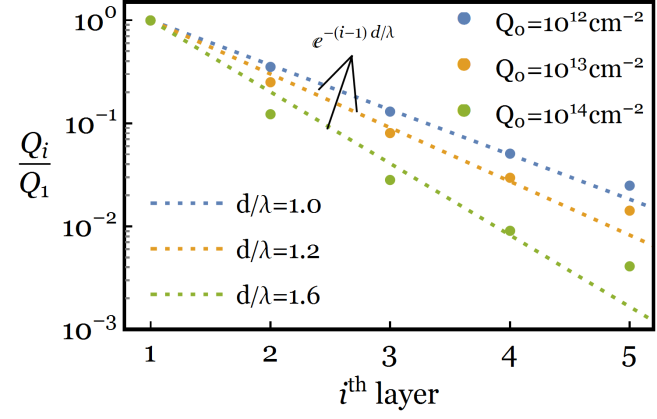 | 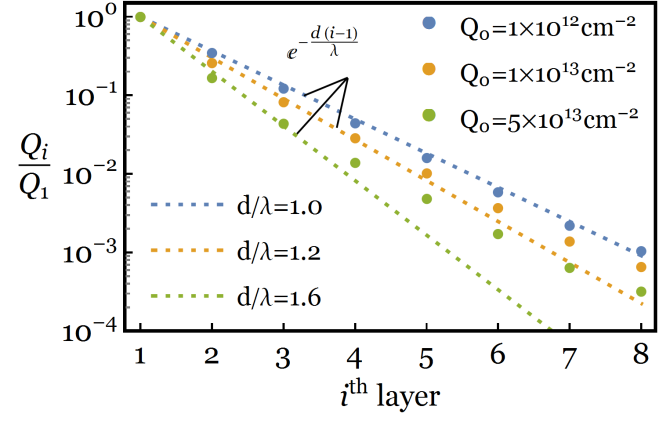 |
| --- | --- |
| **Figure S3.** Normalized average charge profiles across the layers of **(a)** a 5-LG system and **(b)** an 8-LG system for different values of 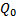. A decay length (*d*/*λ*) of 1.0, 1.2 and 1.6 is found by fitting the data with a function *e*^−(^*^i^*^−1)^*^d/^*^λ^. | |

##

## S5. Temperature-Dependent Discrete Model

In order to propose a mathematical discrete model that can be numerically solved at
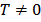
, we had to use a uniform charge distribution model, in which. **Eqs. (2)** and **(4)** in the main text reduce to a constant charge density (
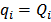
) and a constant Fermi level (
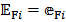
), respectively. Therefore, the charge density of each layer can be expressed by

| 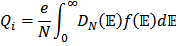 | (S21) |
| --- | --- |

where

| 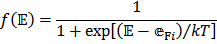 | (S22) |
| --- | --- |

is the Fermi-Dirac distribution function,
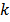
 the Boltzmann constant,
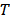
 the absolute temperature, and corresponds to **Eq. (1)** in the main text. Also, the electrostatic energy and band-filling energy at temperature can be given, respectively, by

|  | (S23) |
| --- | --- |

and

|  | (S24) |
| --- | --- |

The charge distribution of *N*-LG is then determined by minimizing the total energy with respect to each of the variational parameters. It is reasonably believed that the above mathematical simplification does not qualitatively and pretty much quantitatively change the results presented in **Fig. (7)** of the main text since our results for obtained by the model given in this section and the one proposed in the main text are very close, if not identical, over the entire range of .

## References

[1] P. Silvestrov and K. Efetov, Charge accumulation at the boundaries of a graphene strip induced by a gate voltage: Electrostatic approach. Phys. Rev. B 77, 155436 (2008).

[2] F. T. Vasko and I. V. Zozoulenko, Conductivity of a graphene stripe: Width and gate-voltage dependencies. Appl. Phys. Lett. 97, 092115 (2010).

[3] Z. Wang, R.W. Scharstein, Electrostatics of graphene: Charge distribution and capacitance. Chemical Physics Letters, 489 (2010);229-236.

[4] A.A.R. Wilmes, S.T. Pinho, A coupled mechanical-charge/dipole molecular dynamics finite element method, with multi-scale applications to the design of graphene nano-devices. International Journal For Numerical Methods In Engineering, 100;2014:243-276.

[5] J. Chae et al., Enhanced Carrier Transport along Edges of Graphene Devices, Nano Letter, 2012, 12 (4), pp 1839–1844.

[6] C. Barraud, T. Choi, P. Butti, I. Shorubalko, T. Taniguchi, K. Watanabe, T. Ihn and K. Ensslin, Field effect in the quantum Hall regime of a high mobility graphene wire. J. Appl. Phys. 116, 073705 (2014).

[7] B.U. Felderhof, Derivation of the love equation for the charge density of a circular plate condenser. arXiv:1309.3662v1; 2013.

[8] J.C. Cooke. The Coaxial circular disc problem. Zeitschrift fur Angewandte Mathematik und Mechanik, 38, 1958;349-356.

[9] [S. Thongrattanasiri](http://scitation.aip.org/content/contributor/AU0108086;jsessionid=RG9PrAEO7Kmyiy004UHdjIgJ.x-aip-live-03), [I. Silveiro](http://scitation.aip.org/content/contributor/AU0282273;jsessionid=RG9PrAEO7Kmyiy004UHdjIgJ.x-aip-live-03) and [F.J.G. de Abajo](http://scitation.aip.org/content/contributor/AU0282274;jsessionid=RG9PrAEO7Kmyiy004UHdjIgJ.x-aip-live-03). Plasmons in electrostatically doped graphene. Appl. Phys. Lett. 100, 201105 (2012).

[10] T. Ohta, A. Bostwick, J.L. McChesney, T. Seyller, K. Horn, E. Rotenberg. Interlayer interaction and electronic screening in multilayer graphene investigated with angle-resolved photoemission spectroscopy. Phys. Rev. Lett. 98, 206802 (2007).

[11] D. Ziegler, P. Gava, J. Güttinger, F. Molitor, L. Wirtz, M. Lazzeri, A. M. Saitta, A. Stemmer, F. Mauri, and C. Stampfer. Variations in the work function of doped single- and few-layer graphene assessed by Kelvin probe force microscopy and density functional theory. Phys. Rev. B 83, 235434 (2011).

[12] X. Wang, J.B. Xu, W. Xie, and J. Du, Quantitative Analysis of Graphene Doping by Organic Molecular Charge Transfer. J. Phys. Chem. C. 2011, 115, 7596-7602.

[13] M. A. Kuroda, J. Tersoff, and Glenn J. Martyna. Nonlinear Screening in Multilayer Graphene Systems, Phys. Rev. Lett. 106, 116804 (2011).

[14] Y. Sui and J. Appenzeller, Screening and Interlayer Coupling in Multilayer Graphene Field-Effect Transistors. Nano Lett., 2009, 9 (8), pp 2973–2977.
